# A Phytase-Based Reporter System for Identification of Functional Secretion Signals in Bifidobacteria

**DOI:** 10.1371/journal.pone.0128802

**Published:** 2015-06-18

**Authors:** Annika Osswald, Christina Westermann, Zhongke Sun, Christian U. Riedel

**Affiliations:** 1 Institute of Microbiology and Biotechnology, University of Ulm, 89068, Ulm, Germany; 2 College of Life Sciences and Agriculture, Zhoukou Normal University, Chuanhui District, 466001, Zhoukou City, P.R. China; Robert Koch-Institute, GERMANY

## Abstract

Health-promoting effects have been attributed to a number of *Bifidobacterium* sp. strains. These effects as well as the ability to colonise the host depend on secreted proteins. Moreover, rational design of protein secretion systems bears the potential for the generation of novel probiotic bifidobacteria with improved health-promoting or therapeutic properties. To date, there is only very limited data on secretion signals of bifidobacteria available. Using *in silico* analysis, we demonstrate that all bifidobacteria encode the major components of Sec-dependent secretion machineries but only *B*. *longum* strains harbour Tat protein translocation systems. A reporter plasmid for secretion signals in bifidobacteria was established by fusing the coding sequence of the signal peptide of a sialidase of *Bifidobacterium bifidum* S17 to the phytase gene *appA* of *E*. *coli*. The recombinant strain showed increased phytase activity in spent culture supernatants and reduced phytase levels in crude extracts compared to the control indicating efficient phytase secretion. The reporter plasmid was used to screen seven predicted signal peptides in *B*. *bifidum* S17 and *B*. *longum* E18. The tested signal peptides differed substantially in their efficacy to mediate protein secretion in different host strains. An efficient signal peptide was used for expression and secretion of a therapeutically relevant protein in *B*. *bifidum* S17. Expression of a secreted cytosine deaminase led to a 100-fold reduced sensitivity of *B*. *bifidum* S17 to 5-fluorocytosine compared to the non-secreted cytosine deaminase suggesting efficient conversion of 5-fluorocytosine to the cytotoxic cancer drug 5-fluorouracil by cytosine deaminase occurred outside the bacterial cell. Selection of appropriate signal peptides for defined protein secretion might improve therapeutic efficacy as well as probiotic properties of bifidobacteria.

## Introduction

Bifidobacteria are an important component of the normal human gastrointestinal microbiota and, besides lactobacilli, the most frequently used microbial supplements in functional foods and probiotic formulations [1]. The concept of functional foods containing live microbial supplements, i.e. probiotics, is based on the observation that some of the commensal bacteria of the human microbiota have beneficial effects in different *in vitro* settings, small animal models or clinical trials [1,2]. These beneficial effects are, in most cases, strain-specific and include maintenance of remission in paediatric ulcerative colitis (UC), prevention of *Clostridium difficile*- and antibiotic-associated diarrhoea, and a reduced mortality in necrotizing enterocolitis (NEC) [2].

Some of the health-promoting effects of probiotics and bifidobacteria seem to be mediated by secreted factors. Several anti-inflammatory factors of bifidobacteria potentially involved in their effects in UC and NEC are secreted proteins. One example is a eukaryotic-type serine protease inhibitor (serpin). Serpin was initially identified in the genome sequence of *B*. *longum* NCC2705 [3] and shown to inhibit pancreatic and neutrophil elastases [4]. Later, serpin-like proteases were also identified in other bifidobacteria [5,6]. Serpin is hypothesized to confer protection against proteolysis by pancreatic elastase in the gastrointestinal environment as well as to inhibit proteolytic damage by neutrophil elastase during intestinal inflammation [4]. A secreted protein of *B*. *animalis* subsp. *lactis* was shown to inhibit inflammatory chemokine secretion by TNF-α challenged cultured intestinal epithelial cells. Similarly, a released peptide factor of *B*. *infantis* was able to prevent loss of epithelial integrity in response to TNF-α or IFN-γ challenge in an *in vitro* setting [7].

The ability to exert a health-promoting effect via secreted proteins depends on an active metabolism and, thus, on acquisition of nutrients. Various strains and species of bifidobacteria were shown to ferment different high molecular weight substrates derived from both the host and its diet including mucus [8], human milk oligosaccharides [9,10], starch [11], and other plant-derived polysaccharides [12]. All these substrates require degradation by extracellular enzymes prior to uptake and further breakdown for energy conservation. The ability to utilize these substrates is thought to provide a selective advantage over other bacteria, aiding the colonization of breast-fed infants and persistence during later stages of life [13–16] and hence support their health-promoting effects.

In addition to their reported health-promoting effects, some strains of bifidobacteria were shown colonise solid tumours in various mouse models [17–20] and thus have gained increasing interest as vectors for delivery of therapeutic genes in cancer therapy [21–23]. The most widely used approach in bacterial tumour targeting is expression of enzymes that convert non-toxic prodrugs to therapeutically active compounds [24]. One example of a well-studied prodrug-converting enzyme (PCE) is cytosine deaminase (CD). This enzyme converts the non-toxic prodrug 5-fluorocytosine to 5-fluorouracil, which interferes with DNA synthesis and thus inhibits proliferation of tumour cells. In order to improve the efficacy of prodrug conversion, while at the same time avoiding inhibitory effects on the proliferation of the delivery vector, PCEs are mostly used as secreted proteins.

Altogether, these findings indicate that secreted proteins might be important for establishment and maintenance of stable bifidobacterial populations in the gastrointestinal tract. Moreover, efficient protein secretion is crucial for functionality of bifidobacteria as probiotics and gene delivery vectors for tumour targeting approaches. In bacteria, the majority of extracellular proteins is secreted by either the Sec or the Twin arginine translocation (Tat) pathway [25,26]. The Sec pathway exports proteins across the cytoplasma membrane in an unfolded state, whereas the Tat-pathway transports folded proteins [27,28]. Both pathways depend on secretion signals usually located in the N-terminus of the substrate that are distinct yet share structural similarities [27].

In bifidobacteria, protein secretion has not been analysed in great detail and there is only one study employing a nuclease reporter to identify bifidobacterial signal sequences [29]. In the present study, we aim at providing a more systematic analysis of protein secretion and associated signal peptides of bifidobacteria, developing a system to analyse these SPs, and devising a tool for efficient expression of extracellular proteins in bifidobacteria.

## Materials and Methods

### Bacterial strains, plasmids and growth conditions

All strains and plasmids used in this study are listed in S1 Table. *E*. *coli* DH10B was used as cloning host and for propagation of plasmids and grown in Luria broth (LB) at 37°C. Bifidobacteria were grown anaerobically at 37°C in Reinforced Clostridial Medium (RCM, BD Difco, Germany) or Lactobacilli MRS (BD Difco, Germany) broth supplemented with 0.5 g/L L-cysteine hydrochloride-monohydrate (MRSc). For cultivation of *E*. *coli* and *Bifidobacterium sp*. strains harbouring plasmids, 100 μg/ml spectinomycin were added to culture media. All media were prepared with ultrapure water.

### Cloning procedures

Genomic DNA of *E*. *coli* DH10B was used as template for amplification of *appA*. Coding sequences of predicted SPs were amplified from genomic DNA of *B*. *bifidum* S17 or *B*. *longum* E18. PCRs were performed using Phusion DNA Polymerase (Thermo Scientific, Germany). All primers used in this study (S2 Table) were purchased from Eurofins Genomics GmbH (Germany). Thermo cycling was performed on a FlexCycler (Analytik Jena, Germany) with annealing temperature optimized for each primer pair. The *appA* gene of *E*. *coli* K-12 encoding a phytase was amplified without its native signal peptide sequence using primers PhytF and PhytR. The obtained PCR product was digested with restriction enzymes *Xho*I and *Hind*III and ligated to the 4,423 bp fragment of similarly digested pMDY23-P_*gap*_ [30], i.e. the vector backbone including P_*gap*_ but lacking the *gusA* gene. This yielded pMgapP, which harbours the *appA* gene fused directly to P_*gap*_ without any signal sequence.

Coding sequences of signal peptides were fused to the *appA* gene by splicing-by-overlap-extension (SOEing) PCR [31]. The coding sequences for different SPs were amplified using a forward primer and a SOEing reverse primer. The PCR was designed to include two additional amino acid residues after the predicted cleavage site to preserve the recognition sequence for cleavage. In parallel, a SOEing forward primer with complementary sequence to the SP reverse primer was used together with primer PhytR for amplification of *appA*. To fuse the SP coding sequence to *appA*, a second round of PCR was performed using the two PCR products of the first round as template, the SP forward primer, and primer PhytR. To increase specificity, DMSO was added to the PCR reaction to a final concentration of 5% (v/v) and annealing temperature was set to 70°C. The obtained PCR products were digested with restriction enzymes *Xho*I and *Hind*III and ligated to the 4,423 bp fragment of *Xho*I/*Hind*III cut pMDY23-P_*gap*_ yielding plasmids with exact translational fusions of the different SPs to AppA.

In order to generate a vector for expression of a secreted cytosine deaminase (CD), the signal peptide of the *bbif_1734* gene encoding a sialidase was amplified from *B*. *bifidum* S17 chromosomal DNA by PCR using primers *SP*_fw_*Sal*I and *SP*_rev_*Hind*III. The *codA* gene was amplified from *E*. *coli* K-12 chromosomal DNA with the primers *codA_*fw*_Hind*III and *codA_*rev*_Sac*II by PCR. Both PCR products were digested with *Hind*III and subsequently joined by a ligase reaction. The fusion was then amplified by PCR using primers *SP*_fw_*Sal*I *codA_*rev*_Sac*II and the product was digested with *Sal*I and *Sac*II and ligated to the 4,423 bp fragment of *Sal*I/*Sac*II cut pMGS-P_*gap*_-*bopA*His_6_ [32] containing P_*gap*_ to yield pAO-S0-CD. The control plasmid pAO-CD, which contains a SP-less CD construct, was obtained by amplifying *codA* with primers *codA_*fw*_Sal*I and *codA_*rev*_Hind*III and ligation of the *Sal*I/*Hind*III digested PCR product to to the 4,423 bp fragment of *Sal*I /*Hind*III cut pMGS-P_*gap*_-*bopA*His_6_.

Following transformation into *E*. *coli*, plasmids of spectinomycin resistant colonies were checked for correct inserts by PCR. Plasmids of positive clones were verified by restriction analysis and Sanger sequencing and constructs with correct sequences were transformed into either *B*. *bifidum* S17 or *B*. *longum* E18 as described elsewhere [33].

### Phytase assay

For phytase samples, special attention is needed to deplete potential free phosphate contamination. All related reagents and medium were prepared in ultrapure ddH_2_O (18 MΩ·cm; Millipore, USA).

Recombinant *B*. *bifidum* S17 or *B*. *longum* E18 strains were grown in 50 ml RCM containing 100 μg/ml spectinomycin under anaerobic conditions. At the indicated time points, 5 ml of the cultures were harvested and centrifuged (5 000 × g, 5 min, 4°C). Supernatants were filter-sterilized and used for determination of extracellular phytase activity. Bacterial pellets were washed twice in 1 ml of 0.2 M sodium citrate buffer, pH 5.5, and resuspended in 500 μl of the same buffer. Bacterial suspensions were transferred to cryotubes containing 250 μg glass beads and disrupted during 2 cycles of 35 s at 6500 rpm in a Precellys 24 homogenisator (PEQLAB Biotechnologie GmbH, Germany). Lysates were centrifuged (13 000 × g, 5 min, 4°C) and the supernatant was retained as crude extracts for determination of intracellular phytase activity. Total protein in crude extracts was quantified using the Pierce BCA protein assay kit (Thermo Scientific, Germany).

Phytase activity in supernatants and crude extracts was quantified using an assay described elsewhere [34] with minor modifications. Briefly, 100 μl sample (supernatant or crude extract) were pre-incubated for 5 min at 37°C and then mixed with 100 μl 10.8 mM sodium phytate (50% (w/w) phytic acid diluted in 0.2 M sodium citrate buffer). The reaction was carried out at 37°C for 15 min, and then stopped by adding 200 μl 15% tricholoroacetic acid (TCA). After centrifugation (14 000 × g, 2 min), an aliquot of 20 μl was mixed with 480 μl ultrapure ddH_2_O and 500 μl color reagent (mix of 1M sulfuric acid, 2.5% (w/v) ammonium molybdate in ddH_2_O, and 10% (w/v) ascorbic acid in ddH_2_O at a ratio of 3:1:1). The mixture was incubated at 50°C for 15 min, and 100 μl were transferred to a transparent 96-well microtiter plate (Thermo Scientific, Germany). Absorbance at 820 nm was measured in triplicate using an Infinite M200 multimode microplate reader (Tecan, Switzerland). Phytase activity equivalents were calculated using to a standard curve of two-fold serial dilutions of a 9 mM potassium dihydrogen phosphate in water. Phytase activity equivalents were defined as the amount of enzyme that catalyses the release of 1 μmol of inorganic phosphate per minute from 5.4 mM sodium phytate (i.e. 100 μl sample + 100 μl 10.8 mM sodium phytate solution, see above) under the conditions of the assay. Activities were expressed as relative phytase units (RPU) per ml in supernatant and RPU/mg in crude extracts.

### Phytate degradation assays

To test for phytate degradation an agar plate assay developed for yeasts [35] was adapted for bifidobacteria using RCM agar (RCA) containing calcium phytate (Ca-phytate) as substrate. RCM phytate agar was prepared by adding 3 g/l calcium carbonate (Sigma, Germany) and 1.5 ml of a 50% (w/w) phytic acid solution in H_2_O (Sigma) to standard RCM agar prior to autoclaving. 2 μl of an overnight culture of the tested recombinant bifidobacteria were spotted onto a freshly prepared agar plate. Following incubation for 48 h under standard conditions, phytate degradation can be observed by clear zones in the otherwise opaque agar around the bacterial spots on the plate.

### Growth inhibition by 5-FC

The effect of the conversion of 5-FC to 5-FU by cytosine deaminase was determined for *B*. *bifidum* S17 wildtype and its isogenic derivatives carrying plasmids pAO-CD or pAO-S0_CD. OD_600_ of overnight cultures grown in MRSc was adjusted to 0.1 in fresh medium containing 5-FC at 5, 1, 0.5, 0.1, 0.05, 0.01, 0.005 or 0.001 mg/ml (final concentration). 200 μl aliquots were pipetted into wells of a 96-well microtiter plate in four technical replicates per strain and concentration. After 24 h of anaerobic incubation at 37°C, OD_600_ was measured using an Infinite M200 multimode reader.

### Bioinformatic analysis

The sequenced and annotated genomes of *B*. *longum* E18 (GenBank accession: CM002287), *B*. *bifidum* S17 (CP002220), *B*. *breve* S27 (CP006716), *B*. *animalis* subsp. *lactis* ATCC27673 (CP003941), *B*. *adolescentis* ATCC15703 (AP009256), and *B*. *dentium* Bd1 (CP001750) were searched for genes encoding Sec proteins. Homologies of the deduced amino acid sequences of bifidobacterial Sec homologues to the respective *E*. *coli* K12-W3110 proteins were calculated using the multiple alignment function of the Basic Local Alignment Search Tool (BLAST, ww.ncbi.nlm.nih.gov/BLAST/).

*B*. *bifidum* S17 proteins with predicted extracellular localization were extracted from the precomputed genome results on the cPSORTdb database (version 3) [36]. The sequences of all proteins were analysed for potential SPs using SignalP Version 4.1 [37], TatP [38], and TATFIND [39].

## Results

### Analysis of protein secretion pathways in bifidobacteria

As a basis to establish a protein secretion reporter in bifidobacteria, the genome sequences of a number of representative *Bifidobacterium sp*. strains were analysed. As expected, all analysed genomes harboured genes for SecY, SecE, and SecG, i.e. the major components of the Sec translocon, and the associated ATPase SecA with reasonable homology to the respective proteins of *E*. *coli* K12-W3110 (Table 1). By contrast, genes for Tat-dependent protein secretion were only found in the genomes of *B*. *longum* E18 (Table 1) and other strains of this species (data not shown).

**Table 1:** Components of the major protein secretion machineries encoded on the genomes of representative *Bifidobacterium sp*.

Species/component	Locus Tag	Size [aa]^a^	Homology^b^	GeneBank/RefSeq Accession
***B*. *longum* E18**				[65]
SecY	BLONG_1793	445	43%	ESV34191.1
SecE	BLONG_2073	75	29%	ESV34434.1
SecG	BLONG_1190	82	25%	ESV33665.1
SecA	BLONG_1273	964	49%	ESV33737.1
TatA	BLONG_0090	96	35%	ESV32709.1
TatB	BLONG_0088	86	35%	ESV32707.1
TatC	BLONG_0089	360	29%	ESV32708.1
***B*. *bifidum* S17**				[66]
SecY	BBIF_1484	444	42%	ADO53689.1
SecE	BBIF_0279	75	31%	ADO52484.1
SecG	BBIF_0980	82	30%	ADO53185.1
SecA	BBIF_1223	960	47%	ADO53428.1
***B*. *breve* S27**				[67]
SecY	BS27_1602	445	43%	AHJ25374.1
SecE	BS27_1723	75	29%	AHJ25486.1
SecG	BS27_0998	82	25%	AHJ24814.1
SecA	BS27_1200	960	46%	AHJ24997.1
***B*. *animalis subsp*. *lactis* ATCC27673**				[68]
SecY	BLAC_02015	449	42%	AGW84625.1
SecE	BLAC_01550	76	30%	AGW84532.1
SecG	BLAC_04320	82	31%	AGW85057.1
SecA	BLAC_05590	974	45%	AGW85303.1
***B*. *adolescentis* ATCC15703**				NC_008618.1
SecY	BAD_0341	457	42%	BAF39122.1
SecE	BAD_0245	75	30%	BAF39026.1
SecG	BAD_0833	100	33%	BAF39614.1
SecA	BAD_1020	958	46%	BAF39801.1
***B*. *dentium* Bd1**				[69]
SecY	BDP_0451	457	42%	ADB09126.1
SecE	BDP_0350	75	30%	ADB09028.1
SecG	BDP_1140	82	33%	ADB09771.1
SecA	BDP_1418	958	46%	ADB10026.1

^a^ protein size in amino acid residues (aa).

^b^ percent identity on amino acid sequence level to the respective homologue of *E*. *coli* K12-W3110.

### Construction of a secretion reporter for bifidobacteria

We next extracted a list of all proteins of *B*. *bifidum* S17 predicted to be localized to the extracellular compartment. The N-terminal 60 residues of all proteins retrieved were analysed *in silico* for potential SPs (Table 2). The SP of a sialidase (BBIF_1734) had the highest PSORTb E-score for extracellular localization and the second highest SignalP D-score for SP prediction. Moreover, the sialidase of *Micromonospora viridifaciens*, a closely related member of the phylum Actinobacteria, has been experimentally confirmed to be secreted into the extracellular environment [40,41]. Thus, the SP of BBIF_1734 was named S0 and selected to develop a secretion reporter using the phytase gene *appA* of *E*. *coli* DH10B.

**Fig 1:**
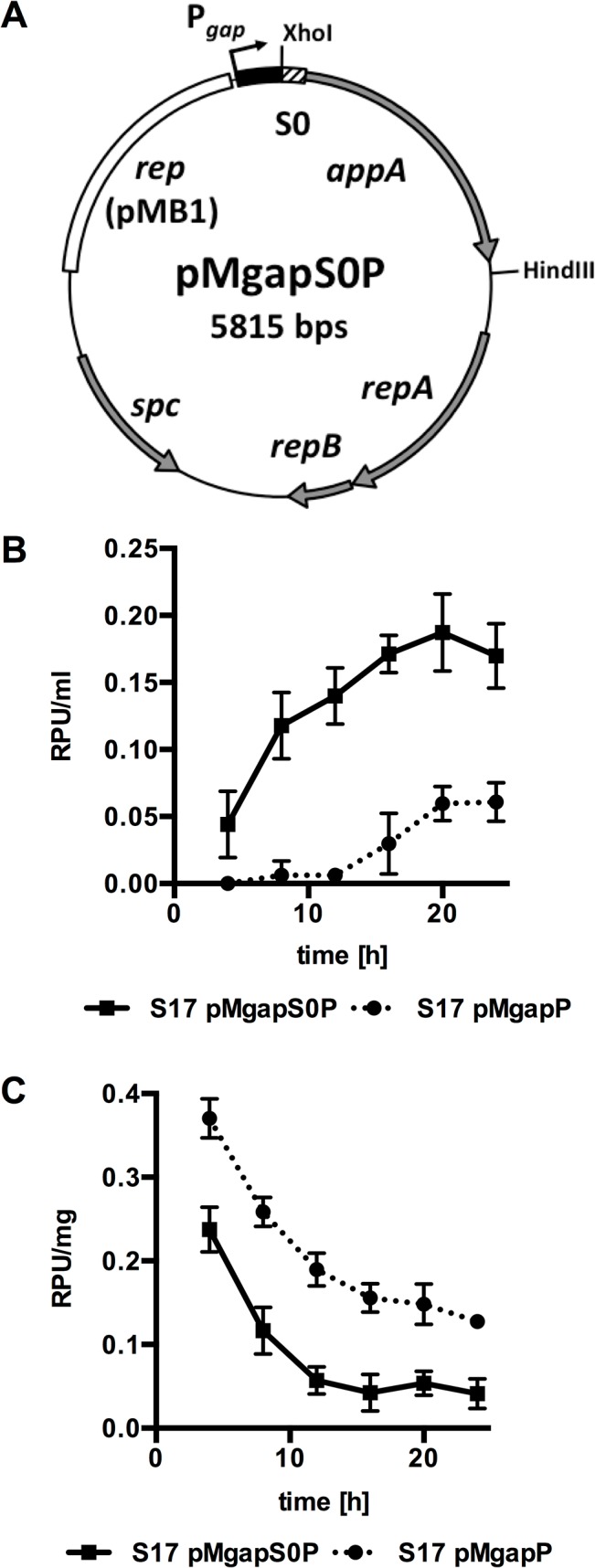
Phytase reporter system to monitor protein secretion by bifidobacteria. (A) Schematic representation of the secretion reporter plasmid pMgapS0P. The vector was constructed by fusing the SP of BBIF_1734 (S0) to the phytase gene *appA* of *E*. *coli* K12 and cloning of the construct under the control of the *gap* promoter (P_*gap*_) of *B*. *bifidum* S17 in the vector backbone of pMDY23-P_*gap*_ using *Xho*I and *Hind*III. Relevant other features are: *rep* (origin of replication for *E*. *coli*), *repAB* (origin of replication for bifidobacteria), *spc* (spectinomycin resistance gene). (B)+(C) Phytase activity in supernatants (B) or crude extracts (C) of *B*. *bifidum* S17/pMgapP (S17 pMgapP) and *B*. *bifidum* S17/pMgapS0P (S17 pMgaS0P) during growth in RCM batch cultures. Values are relative phytase units (RPU) per ml supernatant (B) or mg protein in crude extracts (C) and are mean +/- standard deviation of three independent cultures measured in technical triplicates.

The S0 sequence was fused to the *appA* gene by SOEing PCR and cloned into pMDY23-P_*gap*_ under the control of P_*gap*_, replacing the glucuronidase reporter gene *gusA* (Fig 1A) to yield pMgapS0P. As a control vector, pMgapP was constructed, which harbours an identical *appA* construct fused directly to P_*gap*_ without a signal sequence.

**Table 2:** List of *B*. *bifidum* S17 proteins with predicted extracellular localization and information on the signal peptides identified in their amino acid sequences.

Locus tag	predicted function	PsortB E-score	PsortB SP detection	SignalP D-score
BBIF_0022	Alpha-L-arabinofuranosidase	9.26	+	0.718
BBIF_0048	1,4-beta-N-acetylmuramidase	9.98	+	0.732
BBIF_0108	hypothetical protein BBIF_0108	8.91	-	0.157
BBIF_0158	Trypsin-like serine protease	8.91	-	0.098
BBIF_0246	peptidylprolyl isomerase, FKBP-type	9.26	+	0.556
BBIF_0285	hypothetical protein containing multiple sugar recognition domains	9.76	+	0.886
BBIF_0313	hypothetical protein BBIF_0313	8.91	-	0.191
BBIF_0405	hypothetical protein with CHAP domain	8.91	-	0.408
BBIF_0483	conserved protein with the pectin lyase fold domain	9.13	+	0.723
BBIF_0507	beta-galactosidase BbgIII	7.74	+	0.665
BBIF_0538	hypothetical protein BBIF_0538	8.91	-	0.151
BBIF_1193	serine/cysteine peptidase	9.13	+	0.836
BBIF_1317	alpha-L-fucosidase	9.97	+	0.714
BBIF_1380	hypothetical protein BBIF_1380	9.13	+	0.831
BBIF_1391	D-alanyl-D-alanine carboxypeptidase	9.72	-	0.432
BBIF_1399	hypothetical protein BBIF_1399	8.91	-	0.129
BBIF_1426	hypothetical protein with NlpC/P60 domain	9.73	+	0.633
BBIF_1427	hypothetical protein containing CHAP domain	9.72	-	0.300
BBIF_1457	Rhs family protein	8.91	-	0.134
BBIF_1458	hypothetical protein BBIF_1458	8.91	-	0.125
BBIF_1461	beta-N-acetylglucosaminidase	9.76	+	0.760
BBIF_1576	beta-N-acetylglucosaminidase	9.98	+	0.680
BBIF_1733	sialidase	9.97	+	0.792
**BBIF_1734**	**sialidase**	**9.98**	**+**	**0.862**
BBIF_1740	Alkaline phosphatase	9.73	+	0.776

Both plasmids were transformed into *B*. *bifidum* S17 and phytase activity in crude extracts (intracellular) and culture supernatants (extracellular) of the recombinant strains was measured at various time points during growth. Both strains displayed almost identical growth (data not shown) ruling out any effect of plasmids on growth or phytase activity. Phytase activity markedly increased over time in supernatants of *B*. *bifidum* S17/pMgapS0P (Fig 1B). By contrast, phytase activity in supernatants of the control strain *B*. *bifidum* S17/pMgapP were barely above background until later time points during growth, i.e. stationary growth phase (Fig 1B). On the other hand, phytase activities were higher in crude extracts of *B*. *bifidum* S17/pMgapP than in the strain harbouring pMgapS0P, i.e. the construct with a SP, throughout the experiment (Fig 1C).

### Comparative analysis of various SPs in different *Bifidobacterium sp*. hosts

Following the successful establishment of *appA* as a secretion reporter in *B*. *bifidum* S17, this system was used to test various other bifidobacterial SPs. For this purpose, a total of six SPs with high D-scores according to the SignalP prediction were selected (Table 3). Of these SPs, two belong to proteins from *B*. *bifidum* S17 (BBIF_1681 and BBIF_1761) and two other to proteins from *B*. *longum* E18 (BLONG_1728 and BLONG_0476). Moreover, two SPs of (putative) Tat-secreted proteins of *B*. *longum* E18 (BLONG_0223 and BLONG_1620) were included. To test their functionality, all SPs were fused to the *appA* reporter by SOEing PCR, cloned into pMDY23-P_*gap*_ replacing the *gusA* reporter gene, and the obtained plasmids were introduced into *B*. *bifidum* S17 or *B*. *longum* E18 by electroporation. Monitoring of growth in RCM broth indicated that all recombinant strains show the same growth pattern (data not shown).

**Table 3:** Signal peptides used to establish or tested in the phytase reporter assay and corresponding information on the predicted localization.

Signal peptide	locus tag, predicted function	PsortB E-score ^a^	SignalP D-score ^b^	predicted cleavage site ^c^
**Sec-dependent signal peptides**				
S0	BBIF_1734, sialidase	9.98	0.862	aa 33–37: ASA*AS
S3	BLONG_1728, hypothetical secreted protein with NlpC/P60 domain	3.33	0.891	aa 27–31: ATA*AE
S4	BLONG_0476, conserved hypothetical secreted protein with CHAP domain	3.33	0.838	aa 32–36: AQA*DT
S5	BBIF_1681, subtilisin family peptidase (lactocepin)	9.98	0.798	aa 26–30: ALA*AP
S6	BBIF_1761, surface protein with Gram positive anchor and Cna protein B-type domains	0 ^d^	0.879	aa 29–33: ANA*AD
**Tat-dependent signal peptides**				
S1	BLONG_0223, Tat-secreted glycosidase	4.31	0.865	aa 30–34: AQA*AD
S2	BLONG_1620, putative Tat-secreted pectin lyase-like protein	9.13	0.772	aa 28–32: AFA*QS

^a^ E-score, extracellular score, calculated by cPSORTdb (version 3)

^b^ D-score, discrimination score, calculated by SignalP 4.1.

^c^ amino acid positions (aa), the sequence and the exact cleavage site (*) of the predicted SPs are indicated

^d^ This SP belong to a protein which is predicted to be located in the cell wall. The respective C-score for cell wall localization calculated by PSORTdb was 9.26.

**Fig 2:**
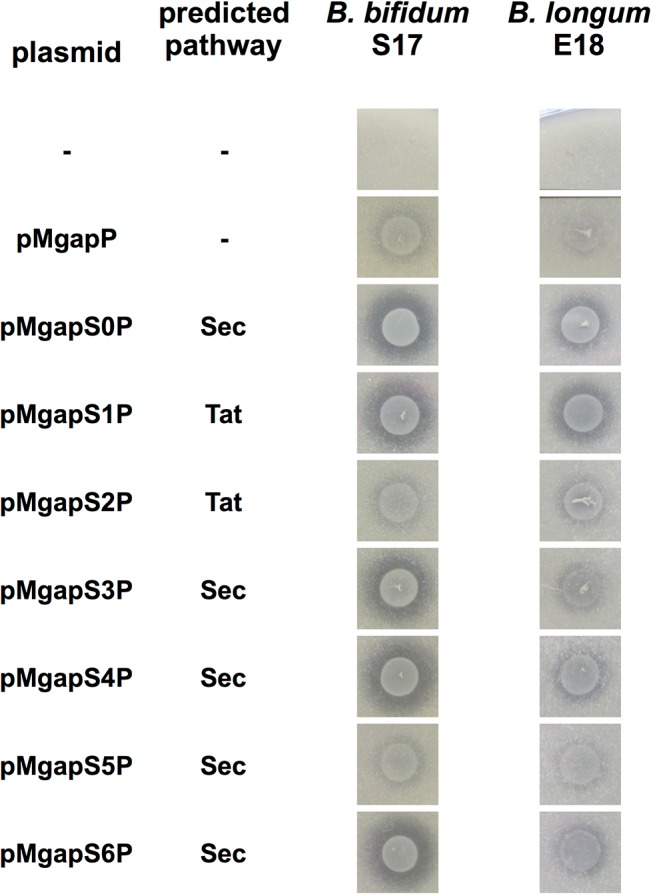
Ca-phytate degradation of recombinant bifidobacteria expressing phytase with different signal peptides. Calcium phytate degradation by recombinant strains of *B*. *bifidum* S17 (A) and *B*. *longum* E18 (B) harbouring pMgapP-derived plasmids containing different SPs (S0-S6). The control plasmid pMgapP contains no SP and serves as a background control for expression of a non-secreted phytase. Overnight cultures of all strains were spotted in triplicate on RCM agar supplemented with 0.15% calcium phytate and imaged after anaerobic incubation for 48 h at 37°C. One representative spot of three independent cultures is shown.

All recombinant strains were analysed for phytase secretion using a phenotypic assay based on the degradation of insoluble Ca-phytate in solid medium (Fig 2). Clear zones of Ca-phytate degradation were observed for *B*. *bifidum* S17 strains harbouring pMgapS0P, pMgapS1P, pMgapS3P, pMgapS4P, and pMgapS6. By contrast, strains harbouring plasmids pMgapS2P and pMgapS5P did not display Ca-phytate degradation above background levels (pMgapP). A similar pattern of Ca-phytate degradation was observed for *B*. *longum* E18 strains, however at somewhat lower levels.

**Fig 3:**
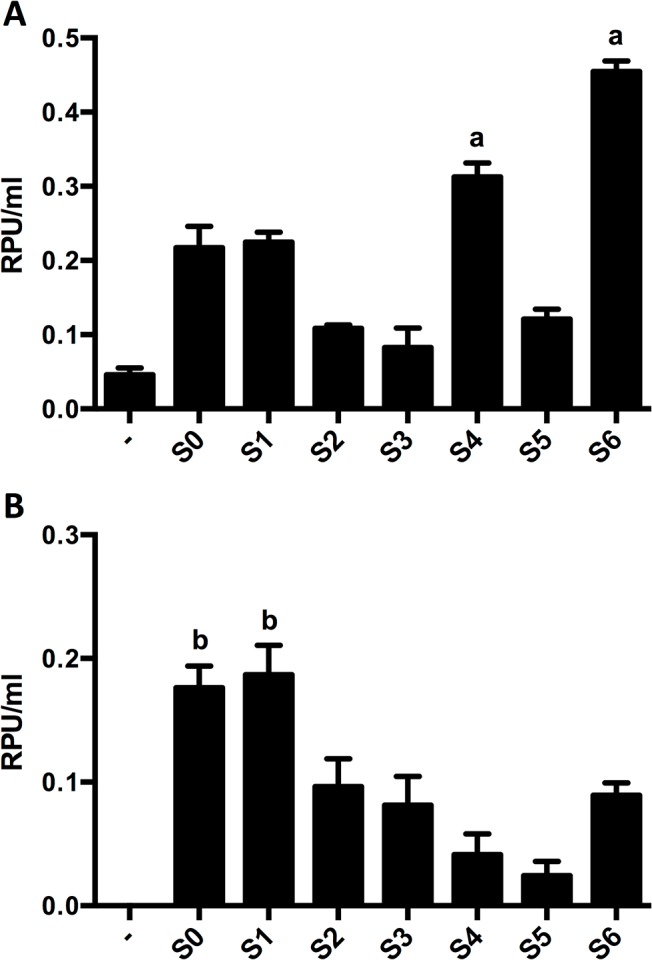
Phytase activity in supernatants recombinant bifidobacteria strains expressing phytase with different signal peptides. Phytase activity was measured in culture supernatants of *B*. *bifidum* S17 (A) or *B*. *longum* E18 (B) harbouring pMgapP-derived plasmids containing different SPs (S0-S6) during growth in RCM batch cultures. The control plasmid pMgapP (-) contains no SP and serves as a background control for expression of a non-secreted phytase. Values are relative phytase units (RPU) per ml supernatant (B) and are mean +/- standard deviation of three independent cultures measured in technical triplicates. Statistical analysis was performed by one-way ANOVA with Bonferroni post-tests for multiple comparisons. Phytase activity in the supernatants of each strain was compared to all other strains. Letters indicate statistical significance of the difference (a: *p* < 0.001 for all comparisons; b: *p* < 0.001 for all comparisons except S0 vs. S1; differences for all other signal peptides were significant at p<0001 for less than 5 other signal peptides). For complete results of the analysis see S3 Table.

In order to get a more quantitative comparison of protein secretion effected by the different SPs, phytase activity was measured in the supernatants of all strains generated. Using S0, highest levels of phytase activity in supernatants of *B*. *bifidum* S17 were observed in stationary growth phase (Fig 1). The sequence upstream of the SP-*appA* constructs including the promoter (P_*gap*_) and all elements relevant for translation, i.e. ribosome binding site, start codon, and the sequence and distance between them, were identical in all strains. Thus, equal levels of expression at least amongst derivatives of the same strain was assumed and samples were collected from cultures grown for 16 h, i.e. early stationary growth phase, for determination of phytase activity. For *B*. *bifidum* S17 strains, highest levels of phytase activity were observed for S6 and somewhat lower yet still efficient secretion was observed when S0, S1 and S4 were used as SP (Fig 3A). Statistical analysis suggests that protein secretion mediated by S4 and S6 is significant different (higher) compared to all other SPs and no difference is observed between S0 and S1 (Fig 3A; S3 Table). In *B*. *longum* E18, S0 and S1 were the most efficient secretion signals (Fig 3B). Again no statistically significant difference between S0 and S1 was observed but both SPs differed from all other SPs (Fig 3B; S3 Table).

### Secretion of cytosine deaminase

**Fig 4:**
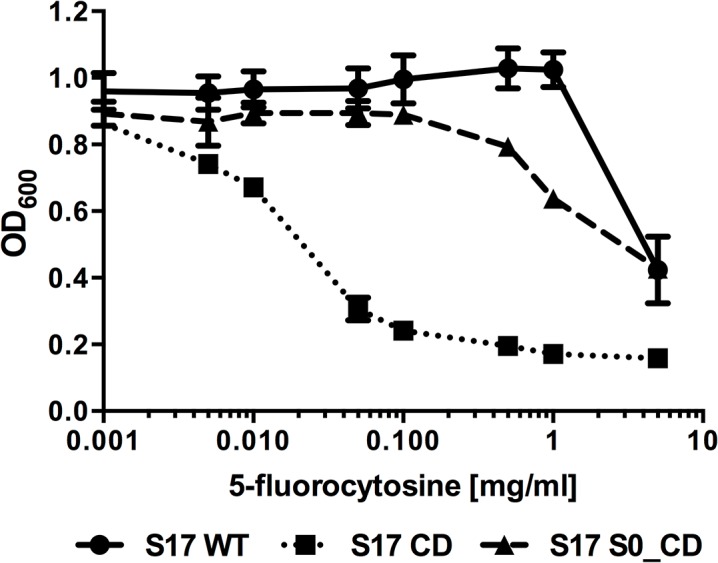
Growth inhibition of recombinant bifidobacteria by 5-fluorocytosine is reduced by secretion of cytosine deaminase. Effect of different concentrations of 5-FC on growth of *B*. *bifidum* S17 wildtype (S17 WT) or isogenic derivatives carrying plasmids pAO-CD (S17 CD) or pAO-S0_CD (S17 S0_CD). Overnight cultures were adjusted to an final optical density at 600 nm (OD_*600*_) of 0.1 in fresh medium containing the indicated concentrations of 5-FC and incubated o/N anaerobically at 37°C and OD_*600*_ was recorded. Values are mean +/- standard deviation of four technical replicates and results of one representative of three independent experiments are shown.

In order to demonstrate applicability of the identified SPs for secretion of a therapeutically relevant protein, we aimed at implementing a PCE approach using CD. For this purpose, we selected S0 based on the efficient secretion of phytase by both *B*. *bifidum* S17 and *B*. *longum* E18. The coding sequence of S0 was fused to the *codA* gene of *E*. *coli* K12. The fusion was cloned under control of P_*gap*_ resulting in pAO-S0_CD, which was transformed into *B*. *bifidum* S17. As a control, *codA* was cloned under control of P_*gap*_ without a SP in the same vector backbone (pAO-CD). Growth of all strains was comparable in the absence of 5-FC (data not shown). Both recombinant strains as well as the wildtype were tested for growth inhibition by the prodrug 5-FC (Fig 4). This revealed that *B*. *bifidum* S17 showed good resistance to the prodrug and growth was only inhibited at the highest prodrug concentration tested (5 mg/ml). Expression of CD without a SP markedly increased sensitivity of the recombinant strain (*B*. *bifidum* S17/pAO-CD). Growth of this strain was already inhibited at 0.005 mg/ml 5-FC. By contrast, growth of *B*. *bifidum* S17/pAO-S0_CD was comparable to that of the wild type in the presence of up to 0.1 mg/ml 5-FC. A slight inhibition of growth was observed only at 5-FC concentrations of 0.5 mg/ml and above but final OD_600_ were higher than that of the strain expressing the SP-less CD at all concentrations tested. Thus, the sensitivity to 5-FC was reduced by expressing CD as a secreted protein by two orders of magnitude suggesting efficient protein export.

## Discussion

A number of traits of bacteria require extracellular proteins. These traits include acquisition of nutrients e.g. by secretion of glycosyl hydrolases for degradation of polysaccharides [42]. Extracellular proteins are not only required for mere survival, but are also involved in the interaction with the host. Thus, survival and interaction with the host of most bacteria crucially depends on secretion of functional proteins. Bifidobacteria as members of the normal human gut microbiota are no exception to this rule. In fact, in the highly competitive environment of the gastrointestinal tract, secretion of proteins might be even more important than in other less densely populated habitats [42].

The vast majority of bacterial proteins are transported by the Sec pathway. Tat-dependent secretion is essential in only a few bacteria [25–27] and was shown to be important for virulence of a wide range of bacterial pathogens [43]. All *Bifidobacterium sp*. genomes analysed harboured genes for a Sec translocon. By contrast, only the *B*. *longum* genomes contained genes for Tat protein export machineries. This is consistent with the conservation and distribution of Sec and Tat systems amongst bacteria.

Various bifidobacterial signal sequences have been used for expression of secreted recombinant proteins. These include the SPs of the galacto-N-biose/lacto-N-biose I-binding protein [44] and exo-xylanase [45–47] of *B*. *longum*, the β-galactosidase of *B*. *bifidum* [48], and Sec2 and ApuB of *B*. *breve* [49,50]. However, in none of these cases the authors provide a rationale for selecting the respective SP and in most cases the sequences have not been analysed. Comparative and systematic studies on secretion systems and signal peptides of bifidobacteria are largely missing. In one study, a nuclease reporter was used to screen a genomic library of *B*. *breve* UCC2003 for signal sequences in *E*. *coli* and subsequent confirmation of positive clones in *L*. *lactis* [29]. This identified three Sec-dependent SPs and three signal sequences of putative membrane proteins. Further analysis of these SPs confirmed that the three Sec-dependent SP are functional for secretion but quantitative analysis of nuclease activity in the supernatants showed no differences in efficiency of protein export.

We used a slightly different approach by selecting SPs of potentially secreted proteins of different bifidobacterial species predicted *in silico*. Using the predicted SPs of the sialidase BBIF_1734, we were able to establish a reporter system for protein secretion in bifidobacteria employing the phytase AppA of *E*. *coli* lacking its native SP. Heterologous expression and secretion of a phytase has previously been demonstrated in lactic acid bacteria [51,52]. The relative levels of extra- and intracellular phytase activity in *B*. *bifidum* S17/pMgapS0P compared to *B*. *bifidum* S17/pMgapP (the strain expressing AppA without SP) suggest that S0 mediates efficient secretion of phytase. Although it is not possible to quantitatively compare levels of intracellular and extracellular levels of phytase due to intrinsic limitations of the Phytex method (RPU/mg of protein in crude extracts vs. RPU/ml supernatant), our results demonstrate that phytase is a valuable reporter system for the identification and analysis of secretion signals in bifidobacteria. The enzyme is resistant against proteases, active in a wide range of pH values, and activity is optimal at pH 4–5, which is the normal pH in stationary phase batch fermentations of bifidobacteria [53].

Bifidobacteria do not encode *appA* homologues since BLAST searches revealed no significant hits in the genus *Bifidobacterium* (data not shown). Nevertheless, phytase activity has been detected in different *Bifidobacterium sp*. [54,55]. Recently, two enzymes of bifidobacteria with phytase activity have been characterized. Based on sequence comparisons, the enzymes belong to a different phylogenetic cluster than the *E*. *coli* AppA enzyme and are more closely related to the phytases of plants, fungi and vertebrates [56]. Intrinsic activity of non-AppA phytases or other phosphatases might explain the slight background observed for *B*. *bifidum* S17/pMgapP.

Using the phytase reporter, we were able to screen a number of other SPs of potentially secreted proteins of *B*. *bifidum* S17 and *B*. *longum* E18. All strains were initially screened for secreted phytase by detection of Ca-phytate degradation in agar plates and measuring phytase activity in culture supernatants. Some of the SPs did not produce zones of phytate degradation on agar, but phytase activity well above background was measured in the supernatants. Thus, Ca-phytate degradation in agar plates is rather a first, but not definite, indicator for the functionality of highly efficient SPs and measuring phytase activity in supernatants allows a more quantitative analysis.

All SPs analysed yielded phytase activities above background in both *B*. *bifidum* S17 and *B*. *longum* E18, suggesting that bifidobacterial SPs might be functional in other *Bifidobacterium* sp. besides their original hosts. Despite their annotation as (putative) Tat substrates, SPs S1 and S2 mediated phytase export in *B*. *bifidum* S17, which lacks a Tat system. Further *in silico* analysis revealed that both SPs yielded no prediction for Tat recognition using TATFIND and no Tat motifs were found by TatP (data not shown). Moreover, S1 and S2 had D-scores of 0.865 and 0.772, respectively, in the SignalP analysis. Collectively, this suggests that the predicted Tat-dependence of these SPs might be false and they might actually be Sec-dependent secretion signals.

Amongst the tested SPs, the SP of BBIF_1734 (S0) mediated efficient protein secretion in both *B*. *bifidum* S17 and *B*. *longum* E18. Thus, we selected S0 to clone a vector for expression and secretion of a therapeutically relevant protein. A number of bacteria including bifidobacteria are investigated as gene delivery vectors for cancer therapy. One of the approaches pursued, is the so-called Bacterial Directed Enzyme Prodrug Therapy (BDEPT), i.e. the use of recombinant bacteria expressing secreted enzymes for conversion of non-toxic prodrugs into their active, cytotoxic form [24]. An enzyme frequently used in BDEPT is the cytosine deaminase [24]. This enzyme is present in a wide range of microorganisms but not in mammalian cells. Its physiological role is deamination of cytosine to uracil and, as a non-specific, non-physiologic side reaction, also catalyses conversion of the prodrug 5-FC to the tumour therapeutic 5-FU.

In a proof-of-principle approach, a construct was generated for expression of a secreted form of the CodA cytosine deaminase of *E*. *coli* in bifidobacteria using the P_*gap*_ promoter and S0 to achieve secretion. *B*. *bifidum* S17/pAO-S0P tolerate doses of 5-FC of 0.2 mg/ml without inhibition of growth. By contrast, growth of the isogenic strain expressing a non-secreted CD is inhibited at concentration of 0.005 mg/ml and above. This demonstrates that a therapeutically relevant protein can be expressed and secreted in its active form using the SP of BBIF_1734. Moreover, the highest systemic concentration of 5-FC that is tolerated during therapy without adverse side effects is 0.1 mg/ml [57]. Thus, expression of CD as a secreted form by *B*. *bifidum* S17 renders this strain tolerant to therapeutically relevant doses.

A number of genetically engineered bifidobacteria expressing various therapeutic genes were successfully used to inhibit tumour growth in mouse models [58–62]. CD has also been expressed in bifidobacteria [63,64] and one of these recombinant strains was successfully used in combination with 5-FC to inhibit growth of a subcutaneous tumours of a melanoma cell line in mice [64]. Expression CD and other proteins in secreted form by bifidobacteria using appropriate secretion signals might improve their therapeutic efficacy.

In summary, our data shows that we have successfully developed a reporter system for identification and analysis of secretion signals in bifidobacteria. We furthermore demonstrated that an SP identified using this report is functional in mediating secretion of a therapeutically relevant protein. Rationally designed systems for secreted proteins might improve the efficacy of recombinant bifidobacteria as probiotic supplements in functional foods or in therapeutic applications such as cancer therapy.

## Supporting Information

S1 Data Amino acid sequences of Sec and Tat homologues of representative *Bifidobacterium sp*. (DOCX)

S1 Table Bacterial strains. (DOCX)

S2 Table Oligonucleotides. (DOCX)

S3 Table Statistical analysis of phytase secretion using different signal peptides. (DOCX)
