# Supplementary material for: A Phytase-Based Reporter System for Identification of Functional Secretion Signals in Bifidobacteria
Source: PLoS One. 2015 Jun 18;10(6):e0128802. doi: 10.1371/journal.pone.0128802 (PMC4472781; doi:10.1371/journal.pone.0128802)
Supplement: S1 Data — (DOCX) [file pone.0128802.s001.docx]

**Supplementary data S4:** Amino acid sequences of representative Sec and Tat homologues of important *Bifidobacterium sp.* of the human microbiota.

***B. longum* E18**

SecG (BLONG_1190):

>gi|560168378|gb|ESV33665.1| Preprotein translocase subunit SecG [Bifidobacterium longum E18] MTALKITLQVIVVLLSFVLTLLILMHKGKGGGLSDMFGGGLTQNAGTSGVAEKNLNRWTVIIALVWVALIIGLDMMTKFNLG

SecE (BLONG_2073):

>gi|560169147|gb|ESV34434.1| Protein translocase subunit SecE [Bifidobacterium longum E18] MAKTSKSEKAVKPNVFMRIGLFIKQIIDELRKVVTPTAKELFFWSLAVFIFVLLLMALVTGMDFGLGKATLWVFG

SecY (BLONG_1793):

>gi|560168904|gb|ESV34191.1| Preprotein translocase, subunit SecY [Bifidobacterium longum E18] MRTLIQALKTKELRKKILFVLFIIIVYRIGSFIPTPGVDYNVVNKCMATIGSASQENFIGLVNLFSGGAMLQLSIFALGVMPYITASIVVQLLRVVIPRFEALHKEGQSGEAKLTQYTRYLTIGLAVLQSTTILVTARSGALFNYQCDQVIPDGSVFNLVVMVLIMTGGTGLIMWMAELVTDKGIGQGMSILIFMSICSGFLPQLWEIGYGTNGTDGDWLKFGIVVGVLVVILIFVDFVELCQRRVPVQYTRRMIGRKMYGGSSTYLPLKINMSGVIPPIFASSILAIPTLIAQFGKSDQSWVKWINANLANTTSVWYIALYALMIVFFCFFYTSITFNPDETADNMKQYGGFIPGIRAGNATSRYLTYVMNRLNTVGAVYLLFVALIPTVLIMALGLNAKLPFGGTTILIIAGVGLDTLRQAKAQTEQFQYTGFLFENVDHKEG

SecA (BLONG_1273):

>gi|560168450|gb|ESV33737.1| Protein translocase subunit SecA [Bifidobacterium longum E18] MVDIVDKALRMGEGHQLKKLENVAKAVNALEDEISALSDEDLKAQTPKFKQEIENGKSLDEIMPEAFATVREVSKRTLGQRHFDVQLMGGAALHWGNIAEMKTGEGKTLVATLPTYLNALEGKGVHVVTVNDYLASYQSELMGRIYRFLGMNVGCIITEQKPPERRKQYNADITYGTNNEFGFDYLRDNMAWEKADLVQRGHHYAIVDEVDSILIDEARTPLIISGPAEGDVTRWYRQFAKLVLKLTRDEDYDVDEKKKVVGILDPGITKVEDFLGIDNLYEPANTALIGYLNNAIKAKELFLRDKDYVVTQGEVLIVDEHTGRILPGRRYNEGLHQAIEAKEGVEVKAENQTFATITLQNYFRMYDKLAGMTGTAETEAAEFMNTYKLGVLPIKTNKPMIRKDQDDLIYRTKKEKLAAIVKDVAKRHAKGQPVLLGTASVESSEVVSTLLDVAKIPHQVLNAKQHEKEAAVVAVAGRKGAVTVATNMAGRGTDIMLGGNVEFLADAKLKSEGYSPEDTPEEYEKRWPGTLNEIKAQVKDEHEEVKELGGLYVLGTERHESRRIDNQLRGRSGRQGDPGESRFYLSLEDDLMRLFNTQLVAQVMAKGMEEGQPIEAKSVTKGVRTAQKAVESRNYEIRKNVLKYDDVMNKQRTVIYSERQAVLKGEDIHKDILRFISDTVESYIKGANKGSEKPKDWDWEGLFKALNTVIPTKVDEDEVRKIVGGLKGAKAVEAVRDLIVEDARQQYGEMEETIGETGLRDLERRVVLAVLDRKWREHLYEMDYLKDGIGLRGMGQRDPLVEYQREGYQMYNSMIEAIKEETVQLLFHIDIKQVATTDEAVDEVEETAESADTIAVASGPDENGESVVEAAEGEVEEEDEDTDAKQAIAESAAASGAGESTLPVAGPAPISHAEGKVPVSKRPKSEELKTPWADGRTFPGTGKNAPCPCGSGRKYKMCHGQNEK

TatA (BLONG_0090):

>gi|560167422|gb|ESV32709.1| Twin-arginine translocation protein TatA [Bifidobacterium longum E18] MPNLRPSELIIILLIIVLLFGAKKLPELAKSIGQSMKIFRAETRTDDPATQQTVNTVSDAPAPAPAAAPAPAPAVPQAVTMNATAAPTTATGAEQR

TatB (BLONG_0088):

>gi|560167420|gb|ESV32707.1| Twin-arginine translocation protein TatB [Bifidobacterium longum E18] MLFGISGTELMVILVIALVLVGPDKLPDYARKVGRFIHSMRVRGQALSEQSNIDVHGLVQDSGINDIRKGLDDATQDVNRLLPPIR

TatC (BLONG_0089):

>gi|560167421|gb|ESV32708.1| Twin-arginine translocation protein TatC [Bifidobacterium longum E18] MSTVPGNPALPAPLAPAMHPGDRDTHLDLDDLIDHSHEATQQASAGAQHPTRRWKRGVGCKSKTRKPRNPDAVMPLADHLVEFRKRFVRAIAGIIIMSIVGWMFSDQVFRILQQPFLTAAGQQQGLMSITFNGVVSAFNVKLEIAFFLGLTASCPWWSYQVWAFINPGLKRKERWTAVTFIGASVPLFLTGAGLAWYLLPQAVAILTGFAPANTATLLSADVYFDFILRMTVAFGLSFLLPVVMAALTMMNAVETRTWLKQWRLATVIAFIFAAVATPTGDPGTLCALALPIIAIYFAAIAVCAVYEHIQLWKVMRAAGEEPKLLKLLHKMRAALPFRAARKAKRTGKQEAPATITAEGE

***B. bifidum* S17**

SecG (BBIF_0980):

>gi|310287501|ref|YP_003938759.1| preprotein translocase subunit SecG [Bifidobacterium bifidum S17] MHILKIVLEIIVVIASILLTLLILMHKGKGGGLSDMFGGGLTQNAGTSGVAEKNLNRWTIFIALVWVAIIIALGLMTKFNLV

SecE (BBIF_0279):

>gi|310286800|ref|YP_003938058.1| preprotein translocase subunit SecE [Bifidobacterium bifidum S17] MAKASKAEKAVKPNVFMRIGLFIKQIIDELRKVVTPTAKELFFWSLGVFIFVVLLMLLVTGMDFGLGKLVLWVFG

SecY (BBIF_1484):

>gi|310288004|ref|YP_003939263.1| protein translocase subunit secY [Bifidobacterium bifidum S17] MRTLIQAFRTKELRNKILFTLAMIIIYRIGSFIPTPGVNYKTVQDCINTLSGSQENFIGLVNLFSGGAMLQLSIFALGVMPYITASIVVQLLRVVIPRFEALHKEGQSGEAKLTQYTRYLTIGLAVLQSTTILVTAQSGALFNNMCSSPIPDNSVWNLSVMVLIMTGGTGLIMWMAELITDKGIGQGMSILIFMSICSGFLPQLWEIGWGTNGTNGDWLKFGIVTGVLVVILVFVDFVELSQRRIPVQYTRRMIGRKMYGGSSTYLPLKINMSGVIPPIFASSILAIPTLIAQFGKSGQSWVVWINKNLANTTSVWYIALYALMIVFFCFFYTSITFNPDETADNMKEYGGFIPGIRAGNATSRYLNYVMNRLNTVGAVYLLFVALIPTVLIMALNLNSKLPFGGTTILIIAGVGLDTLRQAKAQTEQFQYTGFLLEGIDHKEG

SecA (BBIF_1223):

>gi|310287744|ref|YP_003939002.1| protein translocase subunit secA [Bifidobacterium bifidum S17] MVDIVDKALRMGEGHQLKKLENVAKAVNALEDEISALSDEELKGQTAKFKQRLDNGENLDKLMPEAFATVREVSKRTLGQRHFDVQLMGGAALHWGNIAEMKTGEGKTLVATLPSYLNALEGKGVHVVTVNDYLASYQSELMGRIYRFLGMNVGCIITDQKPAERRKQYNADITYGTNNEFGFDYLRDNMAWEKADLVQRGHHYAIVDEVDSILIDEARTPLIISGPAEGDVTRWYRQFARLVPKLTRDEDYEVDEKKKVVGVLDPGITKVEDFLGIDNLYEPSNTALIGYLNNAIKAKELFLRDRDYVVTHGEVLIVDEHTGRILPGRRYNEGLHQALEAKENVEIKAENQTFATITLQNYFRMYDKLAGMTGTAETEAAEFMGTYKLGVLPIPTNKPMIRKDQDDLIFRTKKEKLAAIVKDVAKRHAKGQPVLLGTASVESSEVVSSLLDVAGIDHQVLNAKQHASEAKVVAVAGRKGAVTVATNMAGRGTDIMLGGNVEFLADQKLKSEGYSPEDTPDEYEKRWPGTLAEVKEQVKDEHEEVVELGGLYVLGTERHESRRIDNQLRGRSGRQGDPGESRFYLSLEDDLMRLFNTQLVARVMAKGMPEGEPIEAKSVSKGVRTAQKTVEARNFEIRKNVLKYDDVMNKQRTVIYSERQAVLKGEDIHGDIERFIADTIDSYIKGAQKGSSKPSDWDWDGLFKALKTVFPFELDQDAAKNAADKLKGDKAVAAVRDSLVDQAREEYAELEEKVGEEGLRQLERRVVLAVLDRKWREHLYEMDYLKDGIGLRGMGQRDPLVEYQREGYQMYNSMIEAIKEESVQLLFHVDVQQVSRSEEAGIESDDAAVDEAEEAVGAASSEVDKAEDAGEAETAEESDEKVAIAQSAKESTAGEATAPITGPAPISHAEGKVPANKRPKNEELKTPWSDGRTFPGTSKNAQCPCGSGRKYKMCHGQNEE

***B. breve* S27**

SecG (BS27_0998):

>gi|585136346|gb|AHJ24814.1| Protein translocase subunit secG [Bifidobacterium breve S27] MTALKITLQVIVVLLSFVLTLLILMHKGKGGGLSDMFGGGLTQNAGTSGVAEKNLNRWTVIIALVWVALIIGLDMMTKFNLG

SecE (BS27_1723):

>gi|585137018|gb|AHJ25486.1| Protein translocase subunit secE [Bifidobacterium breve S27] MAKTSTNEKAVKPNVFMRIGLFIKQIIDELRKVVTPTAKELFFWSLAVFIFVLLLMALVTGMDFGLGKATLGIFG

SecY (BS27_1602):

>gi|585136906|gb|AHJ25374.1| Protein translocase subunit secY [Bifidobacterium breve S27]

MRTLIQALKTKELRKKILFVLFIIIVYRIGSFIPTPGVDYNVVNKCMATTGSASQENFIGLVNLFSGGAMLQLSIFALGVMPYITASIVVQLLRVVIPRFEALHKEGQSGEAKLTQYTRYLTIGLAVLQSTTILVTARSGALFNYKCDQVIPDGSVWNLVVMVLIMTGGTGLIMWMAELVTDKGIGQGMSILIFMSICSGFLPQLWEIGYGTNGTDGDWLKFGIVVGVLVVILIFVDFVELCQRRVPVQYTRRMIGRKMYGGSSTYLPLKINMSGVIPPIFASSILAIPTLIAQFGKSDQSWVKWINSNLANTTSVWYIALYALMIVFFCFFYTSITFNPDETADNMKQYGGFIPGIRAGNATSRYLTYVMNRLNTVGAVYLLFVALIPTVLIMALGLNAKLPFGGTTILIIAGVGLDTLRQAKAQTEQFQYTGFLLENVDHKEG

SecA (BS27_1200):

>gi|585136529|gb|AHJ24997.1| Protein translocase subunit secA [Bifidobacterium breve S27] MVDIVDKALRMGEGHQLKKLENVAKAVNALEDEISALSDEDLKAQTPKFKQQIENGKSLDDIMPEAFATVREVSKRTLGQRHFDVQLMGGAALHWGNIAEMKTGEGKTLVATLPTYLNALEGKGVHVVTVNDYLASYQSELMGRIYRFLGMNVGCIITDQKPPERRKQYNADITYGTNNEFGFDYLRDNMAWEKADLVQRGHHYAIVDEVDSILIDEARTPLIISGPAEGDVTRWYRQFAKLVLKLTRDEDYDVDEKKKVVGILDPGITKVEDFLGIDNLYEPANTALIGYLNNAIKAKELFLKDKDYVVTQGEVLIVDEHTGRILPGRRYNEGLHQAIEAKEGVEVKAENQTFATITLQNYFRMYDKLAGMTGTAETEAAEFMNTYKLGVLPIKTNKPMIRKDQDDLIFRTKKEKLAAIVKDVAKRHAKGQPVLLGTASVESSEVVSTLLDVAKIPHQVLNAKQHEKEAAVVAVAGRKGAVTVATNMAGRGTDIMLGGNVEFLADAKLKSEGYSPEDTPEEYEKRWPGTLNEIKAQVKDEHEEVKELGGLYVLGTERHESRRIDNQLRGRSGRQGDPGESRFYLSLEDDLMRLFNTQLVAQVMAKGMEEGQPIEAKSVTKGVRTAQKAVESRNYEIRKNVLKYDDVMNKQRTVIYSERQAVLKGEDIHKDILRFISDTVESYIKGANKGSEKPKDWDWEGLFKALNTVIPTKVDEDEVRKIVGGLKGAKAVEAVRDLIVEDARQQYGEMEETIGETGLRDLERRVVLAVLDRKWREHLYEMDYLKDGIGLRGMGQRDPLVEYQREGYQMYNSMIEAIKEESVQLLFHIDVKQVASTEDAVDEVEESDETADSVTVAAGPDENGESEVEAAEGEVEEEDAKQAIAESAAVSESGESTLPVAGPAPISHAEGKVPASKRPKSDELKTPWADGRTFPGTGKNAPCPCGSGRKYKMCHGQNEK

***B. animalis* subsp. *lactis* ATCC27673**

SecG (BLAC_04320):

>gi|549472332|ref|YP_008606039.1| preprotein translocase subunit SecG [Bifidobacterium animalis subsp. lactis ATCC 27673] MTVVKLILQIVLVVLSLLLTLLILMHKGKGGGLSDMFGGGLTQNAGSSGVAEKNLNRWTVIIALIWVAIIIALGLFTKFGVA

SecE (BLAC_01550):

>gi|549471807|ref|YP_008605514.1| preprotein translocase subunit SecE [Bifidobacterium animalis subsp. lactis ATCC 27673] MARKTDHGEEVIKPNVFMRIGLFIKQIIDELRKVVTPTRKQLFYWSLAVFIFVALLMVFVTAMDFGLGKLSFLIFG

SecY (BLAC_02015):

>gi|549471900|ref|YP_008605607.1| preprotein translocase subunit SecY [Bifidobacterium animalis subsp. lactis ATCC 27673] MRTLIQAFRTKELRNKILFVLGIIIIYRIGSFIPTPGVDYKVVNDCVANTTNNAENFIGLVNLFSGGAMLQLSIFALGVMPYITASIVVQLLRAVIPRFEALHKEGQSGEAKLTQYTRYLTIGLAVLQSTTILVTARSGALFNYQCNNVIPDASIWNMVVMIMVMTGGTGLIMWMAELITEKGIGQGMSILIFLSICSGFLPQLWQIGWGTNGTDGNWTKFAIVTVVLLVIMIFVNYVELSQRRIPVQYTRRMIGRKMYGGSSTYLPLKINMSGVIPPIFASSILAIPTLFAQFGNSQQSWVQWVNKYLANTTSVWYICLYALMIVFFTFFYTEITFNPDETADNMKQFGGFIPGIRAGSATSRYLKYVINRLNTVGAIYLLFVALLPTVLIMALKLNTQLPFGGTTLLIIAGVGLDTLRQAKAQTEQYQYAGFLFENTDHTEGKAIAK

SecA (BLAC_05590):

>gi|549472578|ref|YP_008606285.1| preprotein translocase subunit SecA [Bifidobacterium animalis subsp. lactis ATCC 27673] MVDIVDKALRMGEGRQIKKLEHVAEAVNKLEDQMVVMSDDELKGQTAKFKERLANGETLDDLMPEAFATVREVSKRTLGQRHFDVQLMGGAALHWGNIAEMKTGEGKTLVATLPSYLNALEGKGVHVITVNDYLASYQSELMGRIYRFLGMSVGCIVTGQKPAERRKQYNADITYGTNNEFGFDYLRDNMAWEKNELVQRGHHYAIVDEVDSILIDEARTPLIISGPAEGDVTRWYRQFAKLVLKLNRDEDYEVDEKKKTVGILDPGITKIEDYLGIDNLYEPSNTALIGYLNNAIKAKELFLRDRDYVVTGGEVLIVDEHTGRILPGRRYNEGLHQAIEAKENVEVKAENQTFATITLQNYFRMYDKLAGMTGTAETEAAEFMGTYKLGVLPIPPNKPMIRIDQDDLIFRTKKEKLAAIVKDVAARHRKGQPVLLGTASVESSEVVSSLLDVVEIPHKVLNAKQHEKEAAVVAVAGRKGAVTVATNMAGRGTDIMLGGNVEFLADAELKAKGYSPDDTPEEYEKLWPETLKKIKEQVKDEHEEVKKLGGLYVLGTERHESRRIDNQLRGRSGRQGDPGESRFYLSLEDDLMRLFNTQLVARVMAKGMPEGEPIESKSVSKGVRNAQKAVESRNFEIRKNVLKYDDVMNKQRTVIYSERQAVLKGEDIHEDIEAFISDTLTSYVRGAKNGSDKPADWDWNGLFKAVNDLYPTKVTIDEAKEAAEGLKGDKAVDAVVKLFVDDAEAQYEAFETKLGADGLRTLERRVVLAVLDRKWREHLYEMDYLKDGIGLRGMGQRDPLVEYQREGYQMYNQMIEAIKEETVQLLFHIDLDSIAQTNDNGTDSIDDAAVDSAEIKMGDDVSEDDELNKGNLSEHEPEEAARIDNHADELETAENIAAVKEAAEEGERIPESGLLGPEPMSHAEGKVPARKRPKSEELKTPWSDGRTFPGTPKNAPCPCGSGRKYKMCHGQNEQ

***B. adolescentis* ATCC15703**

SecG (BAD_0833):

>gi|119025851|ref|YP_909696.1| preprotein translocase subunit SecG [Bifidobacterium adolescentis ATCC 15703] MRYYQKQCLRTGGSPVSAMATVKLVLQILLVILSLLLTLLILMHKGKGGGLSDMFGGGLTQNAGSSGVAEKNLNRWTVIIALLWVAIIIALGLMTKFNLI

SecE (BAD_0245):

>gi|119025263|ref|YP_909108.1| preprotein translocase subunit SecE [Bifidobacterium adolescentis ATCC 15703] MAKAKNSEKAIKPNIFMRIGLFIKQIIDELRKVVTPTSKELFFWSLAVFIFVLLLMALVTGMDYGLGKLTLWIFG

SecY (BAD_0341):

>gi|119025359|ref|YP_909204.1| preprotein translocase subunit SecY [Bifidobacterium adolescentis ATCC 15703] MRRCARRWKEPQVRTLIQALKTKELRNKILFTLGIIIIYRIGSFIPTPGVDYTVVQQCVGKMNNASENFIGLVNLFSGGAMLQLSIFALGVMPYITASIVIQLLRVVIPRFEALHKEGQSGEAKLTQYTRYLTIGLAVLQSTTILVTARSGALFNYQCSQVVPDGSVWNLVVMVLIMTGGTGLIMWMAELVTDKGLGQGMSILIFMSICSGFLPQLWEIGWGTNGTDGNWGKFAAVVGTLLVIMILVIYVELAQRRIPVQYTRRMIGRKMYGGSSTYLPLKINMSGVIPPIFASSILAVPTLIAQFGNSDQSWVKWINSNLANTTSVWYIALYALMIVFFCFFYTEITFNPDETADNMKQYGGFIPGIRAGSATSNYLSYVMNRLNTVGAVYLLFVALIPTVLIMALNLNTKLPFGGTTILIIAGVGLDTLRQAKAQTEQFQYAGFLFEDTDHKEGK

SecA (BAD_1020):

>gi|162229910|ref|YP_909883.2| preprotein translocase subunit SecA [Bifidobacterium adolescentis ATCC 15703] MVDIVDKALRMGEGRQIKKLENVAKATNALEDEIAALDDEELKGQTAKFKQRIENGESLDKLMPEAFATVREASKRTLGLRHFDVQLMGGAALHWGNIAEMKTGEGKTLVATLPAYLNALDGQGVHVVTVNDYLASYQAELMGRVYRFLGMSTGCIITNQKPPERRKQYNADITYGTNNEFGFDYLRDNMAWEKSDLVQRGHHYAIVDEVDSILIDEARTPLIISGPAEGDVTRWYRQFARLVLKLNRDEDYEVDEKKKVVGILDPGITKVEDYLGIDNLYEPNNTALIGYLNNAIKAKELFLRDRDYVVTGGEVLIVDEHTGRILPGRRYNEGLHQAIEAKEGVEVKAENQTFATITLQNYFRMYDKLAGMTGTAETEAAEFMGTYKLGVLPIPTNKPMIREDKDDLIFRTKKEKLAAIVRDVAKRHKKGQPVLLGTASVESSEVVSSLLDVAKIPHQVLNAKQHDKEAAVVAVAGRKGAVTVATNMAGRGTDIMLGGNVEFLADAKLKSEGYSPDDTPDEYEKRWPGTLAEIKDQVKDEHEEVVKLGGLYVLGTERHESRRIDNQLRGRSGRQGDPGESRFYLSLEDDLMRLFNTQLVARVMAKGMPEGEPIEAKSVSKGVRTAQKAVESRNFEIRKNVLKYDDVMNKQRTVIYAERQAVLKGADIHEDILKFIDDTVLSYIKGANNGSDKPADWDWDGLFKAISSVYPIAVEQEGAKDAVDKLKGDKAVEALKELIVSDAKDQYSDFEDKLGSEGLRQLERRVVLAVLDRKWREHLYEMDYLKDGIGLRGMGQRDPLVEYQREGYQMYNSMIEAIKEETIQLLFHVDIERVAMTEDEETESDEDEAVNAAEAVMGLDGEAAATGESAPAEPETDDEAEKTTIDELADEQKNEKGIVGMQPISHAEGKVPANKRPKSEELHSPWADGRTFPGTGKNAQCPCGSGRKYKMCHGQNEQ

***B. dentium* Bd1**

SecG (BDP_1140):

>gi|283456031|ref|YP_003360595.1| preprotein translocase subunit SecG [Bifidobacterium dentium Bd1] MAIVKLVLQIVLVIFSLLLTLLILMHKGKGGGLSDMFGGGLTQNAGSSGVAEKNLNRWTVIIALLWVAIIIALGLMTKFNLI

SecE (BDP_0350):

>gi|283455288|ref|YP_003359852.1| protein translocase subunit [Bifidobacterium dentium Bd1] MAKASNREKAVKPNIFMRIGLFIKQIIDELRKVVTPTSKELFFWALAVFIFVLFLMALVTGMDFGLGKLTLWIFG

SecY (BDP_0451):

>gi|283455386|ref|YP_003359950.1| secY protein translocase subunit secY [Bifidobacterium dentium Bd1] MRGRARRWKESQLRTLIQAFKTKELRNKILFTLGIIIIYRVGSFVPTPGVDYTVVQQCVGKMSSTSENFIGLVNLFSGGAMLQLSIFALGVMPYITASIVIQLLRVVIPRFEALHKEGQSGEAKLTQYTRYLTIGLAVLQSTTILVTARSGALFNYQCSQVVPDGSVWNLLVMVLIMTGGTGLIMWMAELITDKGLGQGMSILIFMSICSGFLPQLWEIGWGTKGTDGNWGKFATVVGVLLVIMILVVYVELSQRRIPVQYTRRMIGRKMYGGSSTYLPLKINMSGVIPPIFASSILAIPTLIAQFGNSDQSWVKWINSNLANTTSVWYIALYALMIVFFCFFYTEITFNPDETADNMKQYGGFIPGIRAGSATSRYLNYVMNRLNTVGAVYLLFVALIPTVLIMALNLNTKLPFGGTTILIIAGVGLDTLRQAKAQTEQFQYAGFLFEGTDHQEGK

SecA (BDP_1418):

>gi|283456286|ref|YP_003360850.1| protein translocase subunit secA [Bifidobacterium dentium Bd1] MVDIVDKALRMGEGRQIKKLENVAKATNALEDEIAALNDEELKGQTAKFKERIDNGESLDKIMPEAFATVREASKRTLGLRHFDVQLMGGAALHWGNIAEMKTGEGKTLVATLPAYLNALEGKGVHVVTVNDYLASYQAELMGRVYRFLGMSTGCIITDQKPPERRKQYNADITYGTNNEFGFDYLRDNMSWEKSDLVQRGHHFAIVDEVDSILIDEARTPLIISGPAEGDVTRWYRQFARLVPKLTRDEDYDVDEKKKVVGILDPGITKVEDYLGIDNLYEPNNTALIGYLNNAIKAKELFLRDRDYVVTGGEVLIVDEHTGRILPGRRYNEGLHQAIEAKEGVEVKAENQTFATITLQNYFRMYDKLSGMTGTAETEAAEFMGTYKLGVLPIPTNKPMIREDQDDLIFRTKKEKLAAIVRDVAKRHKKGQPVLLGTASVESSEIVSSLLDVAKIPHQVLNAKQHDKEAAVVAVAGRKGAVTVATNMAGRGTDIMLGGNVEFLADAKLKSEGYSPDDTPDEYEKRWPGTLAEIKEQVKDEHEEVVDLGGLYVLGTERHESRRIDNQLRGRSGRQGDPGESRFYLSLEDDLMRLFNTQLVARVMAKGMPEGEPIEAKSVSKGVRTAQKAVESRNFEIRKNVLKYDDVMNKQRTVIYAERQAVLKGEDIHDDILRFIEDTVTSYIKGANKGSDKPKDWDWEGLFKALASVYPIAVDQDAAKDAVSKLKGDKAVEAVKDLIVADAKDQYLDFEEKLGEEGLRQLERRVVLAVLDRKWREHLYEMDYLKDGIGLRGMGQRDPLVEYQREGYQMYNSMIEAIKEESIQLLFHVDIERVAVTEDTETESDEDEAVNAAEAVMGLEGEAEPTGQSAPAEPETDDEAEKAAIDELAEEHKAEPGIVGMQPISHAEGKVPANKRPKSEELRSPWADGRTFPGTGKNAQCPCGSGRKYKMCHGQNEQ

***E. coli* K12-W3110**

SecG (Y75_P3095):

>gi|388479168|ref|YP_491360.1| preprotein translocase membrane subunit [Escherichia coli str. K-12 substr. W3110] MYEALLVVFLIVAIGLVGLIMLQQGKGADMGASFGAGASATLFGSSGSGNFMTRMTALLATLFFIISLVLGNINSNKTNKGSEWENLSAPAKTEQTQPAAPAKPTSDIPN

SecE (Y75_P3216):

>gi|388479288|ref|YP_491480.1| preprotein translocase membrane subunit [Escherichia coli str. K-12 substr. W3110] MSANTEAQGSGRGLEAMKWVVVVALLLVAIVGNYLYRDIMLPLRALAVVILIAAAGGVALLTTKGKATVAFAREARTEVRKVIWPTRQETLHTTLIVAAVTAVMSLILWGLDGILVRLVSFITGLRF

SecB (Y75_P3565):

>gi|388479630|ref|YP_491824.1| protein export chaperone [Escherichia coli str. K-12 substr. W3110] MSEQNNTEMTFQIQRIYTKDISFEAPNAPHVFQKDWQPEVKLDLDTASSQLADDVYEVVLRVTVTASLGEETAFLCEVQQGGIFSIAGIEGTQMAHCLGAYCPNILFPYARECITSMVSRGTFPQLNLAPVNFDALFMNYLQQQAGEGTEEHQDA

SecY (Y75_P3876):

>gi|388479938|ref|YP_492132.1| preprotein translocase membrane subunit [Escherichia coli str. K-12 substr. W3110] MAKQPGLDFQSAKGGLGELKRRLLFVIGALIVFRIGSFIPIPGIDAAVLAKLLEQQRGTIIEMFNMFSGGALSRASIFALGIMPYISASIIIQLLTVVHPTLAEIKKEGESGRRKISQYTRYGTLVLAIFQSIGIATGLPNMPGMQGLVINPGFAFYFTAVVSLVTGTMFLMWLGEQITERGIGNGISIIIFAGIVAGLPPAIAHTIEQARQGDLHFLVLLLVAVLVFAVTFFVVFVERGQRRIVVNYAKRQQGRRVYAAQSTHLPLKVNMAGVIPAIFASSIILFPATIASWFGGGTGWNWLTTISLYLQPGQPLYVLLYASAIIFFCFFYTALVFNPRETADNLKKSGAFVPGIRPGEQTAKYIDKVMTRLTLVGALYITFICLIPEFMRDAMKVPFYFGGTSLLIVVVVIMDFMAQVQTLMMSSQYESALKKANLKGYGR

SecA (Y75_P0097):

>gi|388476219|ref|YP_488403.1| preprotein translocase subunit, ATPase that targets protein precursors to the SecYE core translocon [Escherichia coli str. K-12 substr. W3110] MLIKLLTKVFGSRNDRTLRRMRKVVNIINAMEPEMEKLSDEELKGKTAEFRARLEKGEVLENLIPEAFAVVREASKRVFGMRHFDVQLLGGMVLNERCIAEMRTGEGKTLTATLPAYLNALTGKGVHVVTVNDYLAQRDAENNRPLFEFLGLTVGINLPGMPAPAKREAYAADITYGTNNEYGFDYLRDNMAFSPEERVQRKLHYALVDEVDSILIDEARTPLIISGPAEDSSEMYKRVNKIIPHLIRQEKEDSETFQGEGHFSVDEKSRQVNLTERGLVLIEELLVKEGIMDEGESLYSPANIMLMHHVTAALRAHALFTRDVDYIVKDGEVIIVDEHTGRTMQGRRWSDGLHQAVEAKEGVQIQNENQTLASITFQNYFRLYEKLAGMTGTADTEAFEFSSIYKLDTVVVPTNRPMIRKDLPDLVYMTEAEKIQAIIEDIKERTAKGQPVLVGTISIEKSELVSNELTKAGIKHNVLNAKFHANEAAIVAQAGYPAAVTIATNMAGRGTDIVLGGSWQAEVAALENPTAEQIEKIKADWQVRHDAVLEAGGLHIIGTERHESRRIDNQLRGRSGRQGDAGSSRFYLSMEDALMRIFASDRVSGMMRKLGMKPGEAIEHPWVTKAIANAQRKVESRNFDIRKQLLEYDDVANDQRRAIYSQRNELLDVSDVSETINSIREDVFKATIDAYIPPQSLEEMWDIPGLQERLKNDFDLDLPIAEWLDKEPELHEETLRERILAQSIEVYQRKEEVVGAEMMRHFEKGVMLQTLDSLWKEHLAAMDYLRQGIHLRGYAQKDPKQEYKRESFSMFAAMLESLKYEVISTLSKVQVRMPEEVEELEQQRRMEAERLAQMQQLSHQDDDSAAAAALAAQTGERKVGRNDPCPCGSGKKYKQCHGRLQ

TatA (Y75_p3342):

>gi|388479414|ref|YP_491606.1| TatABCE protein translocation system subunit [Escherichia coli str. K-12 substr. W3110] MGGISIWQLLIIAVIVVLLFGTKKLGSIGSDLGASIKGFKKAMSDDEPKQDKTSQDADFTAKTIADKQADTNQEQAKTEDAKRHDKEQV

TatB (Y75_p3341):

>gi|388479413|ref|YP_491605.1| TatABCE protein translocation system subunit [Escherichia coli str. K-12 substr. W3110] MFDIGFSELLLVFIIGLVVLGPQRLPVAVKTVAGWIRALRSLATTVQNELTQELKLQEFQDSLKKVEKASLTNLTPELKASMDELRQAAESMKRSYVANDPEKASDEAHTIHNPVVKDNEAAHEGVTPAAAQTQASSPEQKPETTPEPVVKPAADAEPKTAAPSPSSSDKP

TatC (Y75_p3340):

>gi|388479412|ref|YP_491604.1| TatABCE protein translocation system subunit [Escherichia coli str. K-12 substr. W3110] MSVEDTQPLITHLIELRKRLLNCIIAVIVIFLCLVYFANDIYHLVSAPLIKQLPQGSTMIATDVASPFFTPIKLTFMVSLILSAPVILYQVWAFIAPALYKHERRLVVPLLVSSSLLFYIGMAFAYFVVFPLAFGFLANTAPEGVQVSTDIASYLSFVMALFMAFGVSFEVPVAIVLLCWMGITSPEDLRKKRPYVLVGAFVVGMLLTPPDVFSQTLLAIPMYCLFEIGVFFSRFYVGKGRNREEENDAEAESEKTEE

TatE (Y75_p0617):

>gi|388476730|ref|YP_488918.1| TatABCE protein translocation system subunit [Escherichia coli str. K-12 substr. W3110] MGEISITKLLVVAALVVLLFGTKKLRTLGGDLGAAIKGFKKAMNDDDAAAKKGADVDLQAEKLSHKE
