# Supplementary material for: A Phytase-Based Reporter System for Identification of Functional Secretion Signals in Bifidobacteria
Source: PLoS One. 2015 Jun 18;10(6):e0128802. doi: 10.1371/journal.pone.0128802 (PMC4472781; doi:10.1371/journal.pone.0128802)
Supplement: S1 Table — (DOCX) [file pone.0128802.s002.docx]

**Table S1:** Bacterial strains used and generated in the present study.

| **Species/Strain/Plasmid** | **Relevant genotype or description** | **Source/Reference** |
| --- | --- | --- |
| ***E. coli*** |  |  |
| DH10B | Cloning host | Invitrogen |
| ***Bifidobacterium sp.*** |  |  |
| *B. bifidum* S17 | Isolate of a breast-fed infant, genome sequenced | [1] |
| *B. longum* E18 | Isolate of an adult, genome sequenced | [2] |
| *B. bifidum* S17/pMgapP | *B. bifidum* S17 harboring pMgapP | This study |
| *B. bifidum* S17/pMgapS0P | *B. bifidum* S17 harboring pMgapS0P | This study |
| *B. bifidum* S17/pMgapS1P | *B. bifidum* S17 harboring pMgapS1P | This study |
| *B. bifidum* S17/pMgapS2P | *B. bifidum* S17 harboring pMgapS2P | This study |
| *B. bifidum* S17/pMgapS3P | *B. bifidum* S17 harboring pMgapS3P | This study |
| *B. bifidum* S17/pMgapS4P | *B. bifidum* S17 harboring pMgapS4P | This study |
| *B. bifidum* S17/pMgapS5P | *B. bifidum* S17 harboring pMgapS5P | This study |
| *B. bifidum* S17/pMgapS6P | *B. bifidum* S17 harboring pMgapS6P | This study |
| *B. bifidum* S17/pAO-CD | *B. bifidum* S17 harboring pAO-CD | This study |
| *B. bifidum* S17/pAO-S0_CD | *B. bifidum* S17 harboring pAO-S0_CD | This study |
| *B. longum* E18/pMgapP | *B. longum* E18 harboring pMgapP | This study |
| *B. longum* E18/pMgapS0P | *B. longum* E18 harboring pMgapS0P | This study |
| *B. longum* E18/pMgapS1P | *B. longum* E18 harboring pMgapS1P | This study |
| *B. longum* E18/pMgapS2P | *B. longum* E18 harboring pMgapS2P | This study |
| *B. longum* E18/pMgapS3P | *B. longum* E18 harboring pMgapS3P | This study |
| *B. longum* E18/pMgapS4P | *B. longum* E18 harboring pMgapS4P | This study |
| *B. longum* E18/pMgapS5P | *B. longum* E18 harboring pMgapS5P | This study |
| *B. longum* E18/pMgapS6P | *B. longum* E18 harboring pMgapS6P | This study |
| **Plasmids** |  |  |
| pMDY23-P*_gap_* | pMDY23 containing the P*_gap_* promotor of *B. bifidum* S17 for strong constitutive expression in bifidobacteria | [3] |
| pMGS-P*_gap_*-*bopA*His_6_ | pMDY23-P*_gap_* with the *B. bifidum S17* gene *bopA* cloned under control of P*_gap_* | [4] |
| pMgapP | pMDY23-P*_gap_* with the *E. coli* phytase gene *appA* cloned under control of P*_gap_* replacing the gusA reporter gene of pMDY23 | This study |
| pMgapS0P | pMgapP with the coding sequence of the BBIF_1734 signal peptide fused between *appA* and P*_gap_* | This study |
| pMgapS1P | pMgapP with the coding sequence of the BLONG_0223 signal peptide fused between *appA* and P*_gap_* | This study |
| pMgapS2P | pMgapP with the coding sequence of the BLONG_1620 signal peptide fused between *appA* and P*_gap_* | This study |
| pMgapS3P | pMgapP with the coding sequence of the BLONG_1728 signal peptide fused between *appA* and P*_gap_* | This study |
| pMgapS4P | pMgapP with the coding sequence of the BLONG_0476 signal peptide fused between *appA* and P*_gap_* | This study |
| pMgapS5P | pMgapP with the coding sequence of the BBIF_1681 signal peptide fused between *appA* and P*_gap_* | This study |
| pMgapS6P | pMgapP with the coding sequence of the BBIF_1761 signal peptide fused between *appA* and P*_gap_* | This study |
| pAO-CD | pMgapP-derivative for P*_gap_*-driven expression of codA | This study |
| pAO-S0_CD | pMgapP-derivative for P*_gap_*-driven expression of a fusion of the coding sequence of the BBIF_1734 to codA | This study |

1. Zhurina D, Zomer A, Gleinser M, Brancaccio VF, Auchter M, et al. (2011) Complete genome sequence of Bifidobacterium bifidum S17. J Bacteriol 193: 301–302. Available: http://www.ncbi.nlm.nih.gov/pubmed/21037011.

2. Zhurina D, Dudnik A, Waidmann MS, Grimm V, Westermann C, et al. (2013) High-Quality Draft Genome Sequence of Bifidobacterium longum E18, Isolated from a Healthy Adult. Genome Announc 1: pii: e01084–13. Available: http://www.ncbi.nlm.nih.gov/pubmed/24356845. Accessed 8 April 2014.

3. Grimm V, Gleinser M, Neu C, Zhurina D, Riedel CU (2014) Expression of fluorescent proteins in bifidobacteria for analysis of host-microbe interactions. Appl Environ Microbiol 80: 2842–2850. Available: http://www.ncbi.nlm.nih.gov/pubmed/24584243. Accessed 14 May 2014.

4. Gleinser M, Grimm V, Zhurina D, Yuan J, Riedel CU (2012) Improved adhesive properties of recombinant bifidobacteria expressing the Bifidobacterium bifidum-specific lipoprotein BopA. Microb Cell Fact 11: 80. Available: http://www.ncbi.nlm.nih.gov/pubmed/22694891.
