# Supplementary material for: A Phytase-Based Reporter System for Identification of Functional Secretion Signals in Bifidobacteria
Source: PLoS One. 2015 Jun 18;10(6):e0128802. doi: 10.1371/journal.pone.0128802 (PMC4472781; doi:10.1371/journal.pone.0128802)
Supplement: S2 Table — (DOCX) [file pone.0128802.s003.docx]

**Table S2:** Oligonucleotides used in this study. Recognition sequences of relevant restriction enzymes are underlined. Complementary sequences of primers used for SOEing-PCR are highlighted in grey.

| **Name** | **Sequence (3'--> 5')** | **Purpose** |
| --- | --- | --- |
| PMF | GCATATGACTAGTGAGCTC | Colony PCR |
| PMR | AGGACGTAAGTCGACATG |  |
| PhyF | GGCCTCGAG**ATG**CAGAGTGAGCCGGAGCTGA | amplification of *appA* |
| PhyR | CCCAAGCTTAGCCTCAGAGCATTCAGGTAAC |  |
| S0F | GGCCTCGAG**ATG**GTTCGTTCGACCAAGCCATCGCT | amplification of S0 and fusion to appA by SOEing PCR (with primer PhyR) |
| S0R | TCAGCTCCGGCTCACTCTGGCTGGCCGCACTCGCGGTGGATA |  |
| P0F | TATCCACCGCGAGTGCGGCCAGCCAGAGTGAGCCGGAGCTGA |  |
| S1F | GGCCTCGAG**ATG**CATCAATCAACACGAAAGCGGTG | amplification of S1 and fusion to appA by SOEing PCR (with primer PhyR) |
| S1R | TCAGCTCCGGCTCACTCTGATCGGCTGCCTGCGCGGT |  |
| P1F | ACCGCGCAGGCAGCCGATCAGAGTGAGCCGGAGCTGA |  |
| S2F | GGCCTCGAG**ATG**ACATCCCGTCAGGGCAGA | amplification of S2 and fusion to appA by SOEing PCR (with primer PhyR) |
| S2R | TCAGCTCCGGCTCACTCTGTGACTGCGCGAACGCAGC |  |
| P2F | GCTGCGTTCGCGCAGTCACAGAGTGAGCCGGAGCTGA |  |
| S3F | GGCCTCGAGATGAAGACCAAAACTGTAGCTTCT | amplification of S3 and fusion to appA by SOEing PCR (with primer PhyR) |
| S3R | TCAGCTCCGGCTCACTCTGCTCAGCTGCAGTTGCTGTAG |  |
| P3F | CTACAGCAACTGCAGCTGAGCAGAGTGAGCCGGAGCTGA |  |
| S4F | GGCCTCGAGATGACGAACGTACGTGTGATCAA | amplification of S4 and fusion to appA by SOEing PCR (with primer PhyR) |
| S4R | TCAGCTCCGGCTCACTCTGGGTGTCTGCCTGGGCAGG |  |
| P4F | CCTGCCCAGGCAGACACCCAGAGTGAGCCGGAGCTGA |  |
| S5F | GGCCTCGAGATGGCCAATAAGCAATGGCCTCGCTG | amplification of S5 and fusion to appA by SOEing PCR (with primer PhyR) |
| S5R | TCAGCTCCGGCTCACTCTGCGGAGCCGCGAGCGCGGTA |  |
| P5F | TACCGCGCTCGCGGCTCCGCAGAGTGAGCCGGAGCTGA |  |
| S6F | GGCCTCGAGATGAAATCACTGATGAAAAAGGTTTTCGC | amplification of S6 and fusion to appA by SOEing PCR (with primer PhyR) |
| S6R | TCAGCTCCGGCTCACTCTGATCCGCTGCGTTGGCCGT |  |
| P6F | ACGGCCAACGCAGCGGATCAGAGTGAGCCGGAGCTGA |  |
| codA_fw_SalI | GACTACGTCGACATGTCGAATAACGCTTTAC | amplification of *codA* for cloning of pAO-CD |
| codA_rev_HindIII | TACAAGCTTTCAATGATGATGATGATGATGACGTTTGTAATCGATGG |  |
| SP_fw_SalI | GACTAC*GTCGAC*ATGGTTCGTTCGACC | amplification of S0 for cloning of pAO-S0_CD |
| SP_rev_HindIII | GACTACAAGCTTGCTGGCCGCACTCGC |  |
| codA_fw_HindIII | GACTACAAGCTTATGTCGAATAACGCTTTAC | amplification of *codA* for pAO-S0_CD |
| codA_rev_SacII | TACCCGCGGTCAATGATGATGATGATGATGACGTTTGTAATCGATGG |  |
