# Supplementary material for: A Phytase-Based Reporter System for Identification of Functional Secretion Signals in Bifidobacteria
Source: PLoS One. 2015 Jun 18;10(6):e0128802. doi: 10.1371/journal.pone.0128802 (PMC4472781; doi:10.1371/journal.pone.0128802)
Supplement: S3 Table — (DOCX) [file pone.0128802.s004.docx]

**Table S3**: Statistical analysis of the relative efficacies of phytase secretion by *B. bifidum* S17 and *B. longum* E18 strains harbouring plasmids with different signal peptides. Analysis was performed by one-way ANOVA with Bonferroni post-tests for multiple comparisons. Indicated are levels of statistical significance of the difference in phytase activities in the supernatants of the compared strains.

| ***B. bifidum* S17** | | | | | | | | |
| --- | --- | --- | --- | --- | --- | --- | --- | --- |
| **Signal peptide** | **-** | **S0** | **S1** | **S2** | **S3** | **S4** | **S5** | **S6** |
| **-** | **x** | ***p*<0.001** | ***p*<0.001** | ***p*<0.05** | **n.s.** | ***p*<0.001** | ***p*<0.01** | ***p*<0.001** |
| **S0** | ***p*<0.001** | **x** | **n.s.** | ***p*<0.001** | ***p*<0.001** | ***p*<0.001** | ***p*<0.001** | ***p*<0.001** |
| **S1** | ***p*<0.001** | **n.s.** | **x** | ***p*<0.001** | ***p*<0.001** | ***p*<0.001** | ***p*<0.001** | ***p*<0.001** |
| **S2** | ***p*<0.05** | ***p*<0.001** | ***p*<0.001** | **x** | **n.s.** | ***p*<0.001** | **n.s.** | ***p*<0.001** |
| **S3** | **n.s.** | ***p*<0.001** | ***p*<0.001** | **n.s.** | **x** | ***p*<0.001** | **n.s.** | ***p*<0.001** |
| **S4** | ***p*<0.001** | ***p*<0.001** | ***p*<0.001** | ***p*<0.001** | ***p*<0.001** | **x** | ***p*<0.001** | ***p*<0.001** |
| **S5** | ***p*<0.01** | ***p*<0.001** | ***p*<0.001** | **n.s.** | **n.s.** | ***p*<0.001** | **x** | ***p*<0.001** |
| **S6** | ***p*<0.001** | ***p*<0.001** | ***p*<0.001** | ***p*<0.001** | ***p*<0.001** | ***p*<0.001** | ***p*<0.001** | **x** |
| ***B. longum* E18** | | | | | | | | |
| **Signal peptide** | **-** | **S0** | **S1** | **S2** | **S3** | **S4** | **S5** | **S6** |
| **-** | **x** | ***p*<0.001** | ***p*<0.001** | ***p*<0.001** | ***p*<0.001** | ***p*<0.05** | **n.s.** | ***p*<0.001** |
| **S0** | ***p*<0.001** | **x** | **n.s.** | ***p*<0.01** | ***p*<0.001** | ***p*<0.001** | ***p*<0.001** | ***p*<0.001** |
| **S1** | ***p*<0.001** | **n.s.** | **x** | ***p*<0.001** | ***p*<0.001** | ***p*<0.001** | ***p*<0.001** | ***p*<0.001** |
| **S2** | ***p*<0.001** | ***p*<0.01** | ***p*<0.001** | **x** | **n.s.** | ***p*<0.05** | ***p*<0.01** | **n.s.** |
| **S3** | ***p*<0.001** | ***p*<0.001** | ***p*<0.001** | **n.s.** | **x** | **n.s.** | ***p*<0.05** | **n.s.** |
| **S4** | ***p*<0.05** | ***p*<0.001** | ***p*<0.001** | ***p*<0.05** | **n.s.** | **x** | **n.s.** | **n.s.** |
| **S5** | **n.s.** | ***p*<0.001** | ***p*<0.001** | ***p*<0.01** | ***p*<0.05** | **n.s.** | **x** | ***p*<0.05** |
| **S6** | ***p*<0.001** | ***p*<0.001** | ***p*<0.001** | **n.s.** | **n.s.** | **n.s.** | ***p*<0.05** | **x** |
